# *In vivo* dynamics of AAV-mediated gene delivery to sensory neurons of the trigeminal ganglia

**DOI:** 10.1038/s41598-017-01004-y

**Published:** 2017-04-19

**Authors:** Chung H. Dang, Martine Aubert, Harshana S. De Silva Feelixge, Kurt Diem, Michelle A. Loprieno, Pavitra Roychoudhury, Daniel Stone, Keith R. Jerome

**Affiliations:** 1 Vaccine and Infectious Disease Division, Fred Hutchinson Cancer Research Center, Seattle, WA USA; 2 Department of Laboratory Medicine, University of Washington, Seattle, WA USA; 3 Department of Microbiology, University of Washington, Seattle, WA USA; 4 Department of Neuroscience, Columbia University, New York, NY USA

## Abstract

The ability to genetically manipulate trigeminal ganglion (TG) neurons would be useful in the study of the craniofacial nervous system and latent alphaherpesvirus infections. We investigated adeno-associated virus (AAV) vectors for gene delivery to the TG after intradermal whiskerpad delivery in mice. We demonstrated that AAV vectors of serotypes 1, 7, 8, and 9 trafficked from the whiskerpad into TG neurons and expressed transgenes within cell bodies and axons of sensory neurons in all three branches of the TG. Gene expression was highest with AAV1, and steadily increased over time up to day 28. Both constitutive and neuronal-specific promoters were able to drive transgene expression in TG neurons. Levels of vector genomes in the TG increased with input dose, and multiple transgenes could be co-delivered to TG neurons by separate AAV vectors. In conclusion, AAV1 vectors are suitable for gene delivery to TG sensory neurons following intradermal whiskerpad injection.

## Introduction

In vertebrates, the tactile, proprioceptive, and nociceptive afferent neurons of the peripheral nervous system that innervate the face all emerge from a tissue called the trigeminal ganglion (TG). Sensory neurons on each side of the face project from the left or right TG, which are located near the brain and contain thousands of sensory neuron cell bodies. The primary role of the TG is to relay craniofacial sensory and motor nervous stimuli into the central nervous system (CNS) via a large sensory root and a small motor root that enter the brainstem at the level of the pons. The ability to genetically manipulate sensory neurons within the TG would be highly beneficial in the study of neuropathic pain or peripheral nerve injury. Furthermore, gene delivery to sensory neurons of the TG could be used in the development of new treatments for recurrent alphaherpesvirus infections caused by herpes simplex virus (HSV) and varicella zoster virus (VZV), which reside in neuronal cell bodies of the TG during viral latency^1–7^. Thus, the capability to manipulate sensory TG neurons through overexpression of therapeutic genes or inhibitors such as shRNAs would be extremely valuable. One way to genetically manipulate sensory neurons is to use viral vectors as gene transfer agents.

Recombinant viral vectors based on adeno-associated virus (AAV) have been extensively utilized for over 30 years in basic and clinical research requiring efficient gene transfer^8, 9^. AAV vectors are popular due to their ease of manipulation, low immunogenicity, wide tissue tropism and seeming lack of toxicity. They have successfully been used to transduce many different cell types and tissues. Importantly, several AAV serotypes are able to efficiently transduce neurons *in vitro*, and AAV has been extensively used *in vivo* for gene transfer to both the central and peripheral nervous systems (For reviews see refs 10 and 11).

AAV vectors have been studied for gene delivery to sensory nerves of the dorsal root ganglion (DRG) *in vitro* ^12^ or *in vivo* ^13–21^ following intrathecal delivery or direct injection into the sciatic nerve or DRG. Gene expression in DRG neurons has been achieved using AAV vectors based on serotypes 1, 2, 3, 4, 5, 6, 8, 8.2, 9 and rh20 in mice, rats, and pigs. Widespread expression in sensory neurons can be achieved throughout the DRG following AAV delivery, although the expression levels vary depending on the AAV serotype, dose and delivery route. Since the DRG and TG are highly analogous tissues, each containing clusters of cell bodies belonging to sensory afferent neurons, we hypothesized that AAV could also be used to genetically manipulate sensory neurons within the TG with high efficiency.

Due to its anatomical location next to the brain the direct delivery of AAV vectors into the TG is challenging. Therefore, efficient, indirect delivery via a distal peripheral site would be advantageous. A number of AAV serotypes are able to undergo retrograde transport from nerve termini to cell bodies in both CNS and PNS neurons^22–24^. We therefore reasoned that AAV could travel to the TG along craniofacial sensory nerves through a similar process. Here we show that the injection of AAV into the dermis of the whiskerpad, a peripheral site of maxillary sensory nerve endings in rodents, results in efficient transduction of sensory neurons within the mouse TG. AAV vectors derived from serotypes 1, 7, 8, & 9 were all able to express transgenes in neurons of the TG following intradermal whiskerpad delivery, albeit at different levels. Co-delivery of two transgenes could be achieved using two separate AAV vectors. Injection of AAV serotype 1 (AAV1) led to 25% of neuronal cell bodies expressing the reporter transgene within all branches of the TG. This non-invasive AAV delivery route for TG delivery should prove highly useful in future studies of neuropathic pain, peripheral nerve injury, or latent herpesvirus infections.

## Results

### AAV traffics to the trigeminal ganglion after intradermal injection of the mouse whiskerpad

**Figure 1:**
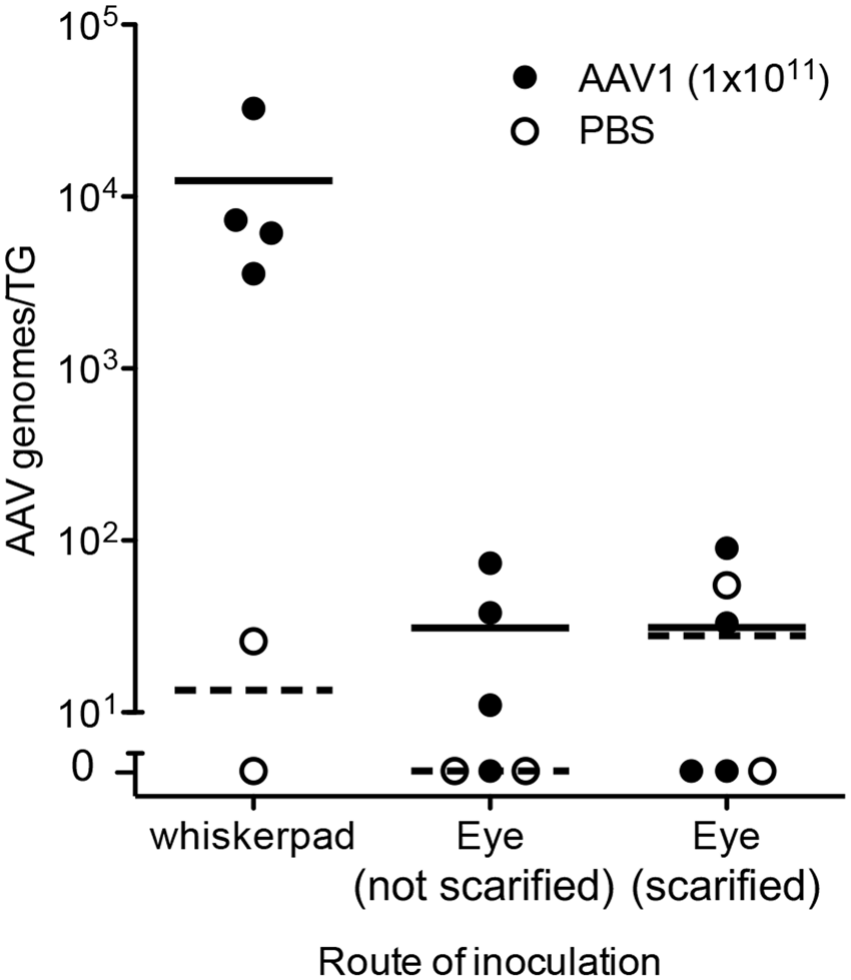
Comparison of ocular and whiskerpad delivery routes for gene delivery to trigeminal ganglia. Swiss Webster mice were inoculated with PBS or scAAV1-smCBA-mCherry vector at a dose of 1 × 10^11^ vector genomes per delivery site. For whiskerpad delivery AAV was delivered by intradermal injection in a volume of 50 μl. For ocular delivery AAV was delivered in a volume of 5 μl onto the eye of mice with non-scarified or scarified corneas. At 7 days post AAV delivery DNA was isolated from trigeminal ganglia and total levels of AAV vector genomes per ganglion were quantified by qPCR. Each circle represents a single trigeminal ganglion (TG) from 2 experimental animals (4 TGs total) per route for AAV1 and 1 animal (2 TGs) per route for PBS control.

In humans, viruses such as HSV-1 that infect sensory neurons in the TG primarily do so by entering the nerve terminals of the ophthalmic, maxillary or mandibular nerves (V1, V2 and V3) that innervate the face, including areas around the eye, nose and mouth. In rodents it is well established that HSV-1 infections of the TG can be established after scarification of both the eye and the whiskerpad followed by HSV-1 exposure^25, 26^. Based on this knowledge we hypothesized that AAV delivery to the scarified eye or by direct injection into the dermis of the whiskerpad might lead to efficient gene delivery to the TG. To determine the most efficient route of delivery for mouse TG transduction we performed a pilot study using self-complementary AAV1 containing the mCherry gene driven by a shortened CBA promoter (scAAV1-smCBA-mCherry) at a dose of 1 × 10^11^ vector genomes per site. We compared inoculation of AAV vectors onto scarified and un-scarified cornea of mice with intradermal injection of AAV into the whiskerpad. At 7 days post scAAV1-smCBA-mCherry exposure, quantitative PCR (qPCR) analysis of vector genome levels was performed using DNA isolated from mouse TG. We found that AAV vector genomes could be readily detected in the TG following whiskerpad injection, but were undetectable or only present at low levels in mice that underwent inoculation through the eye (Fig. 1). Statistical analysis indicated a significant difference between groups which have received AAV (one-way Anova, p = 0.0006) and that the delivery through whiskerpad injection was significantly higher than the eye route with (p = 0.002) or without (p = 0.001) scarification (Post-hoc comparison using the Tukey HSD test). Since intradermal injection of the whiskerpad proved to be the most effective route of AAV administration, it was used for all further experiments.

### Gene delivery to neurons of the trigeminal ganglion is most efficient with AAV1

**Figure 2:**
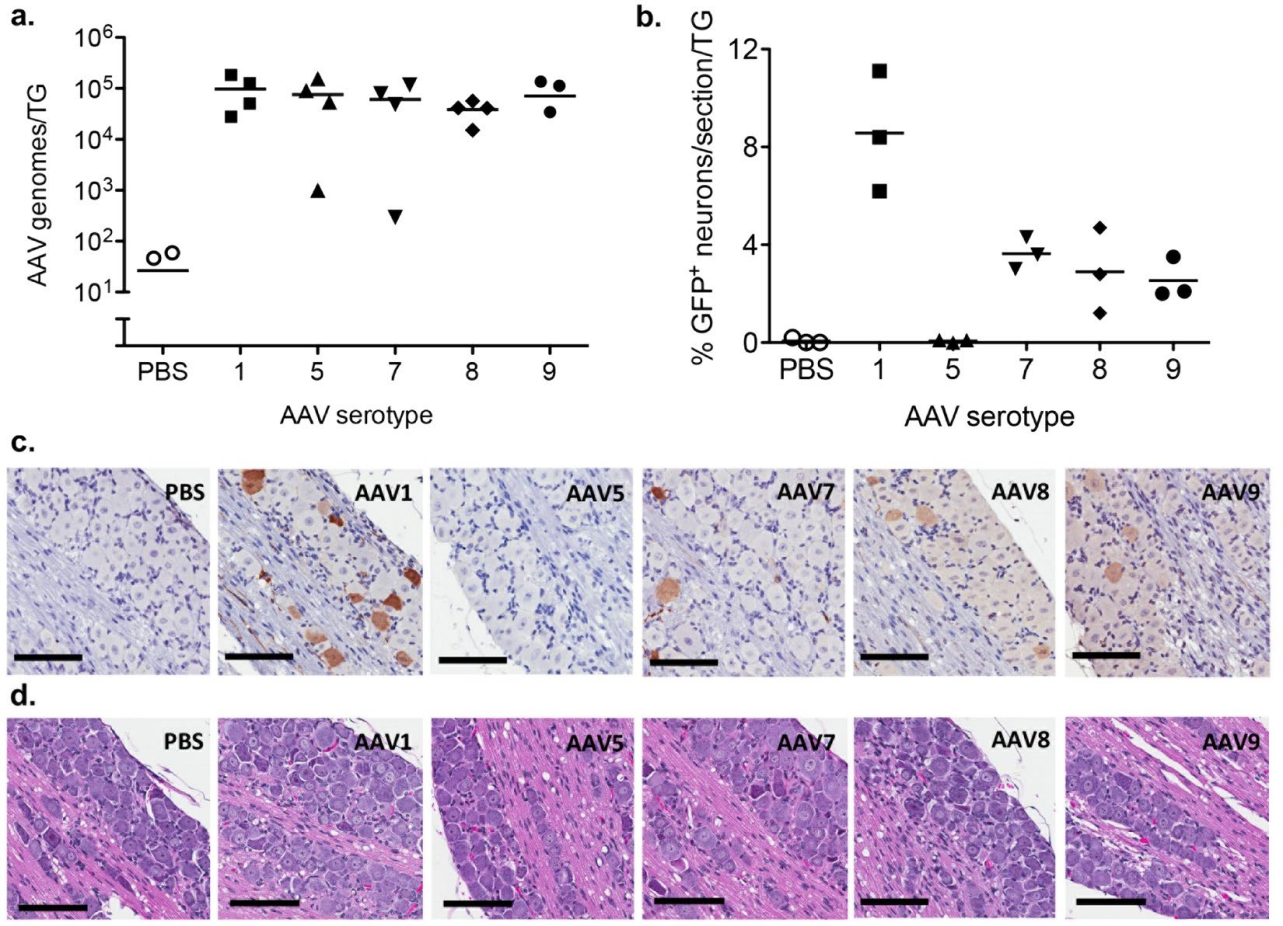
Comparison of AAV serotypes for gene delivery to trigeminal ganglia. Swiss Webster mice were inoculated by intradermal injection of the whiskerpad with PBS or scAAV-smCBA-GFP vectors packaged into AAV serotypes 1, 5, 7, 8 or 9 at a dose of 1 × 10^11^ vector genomes per whiskerpad. (**a**) At 7 days post AAV delivery DNA was isolated from trigeminal ganglia and total levels of AAV vector genomes per ganglion were quantified by qPCR. Each symbol represents one trigeminal ganglion (TG) from a total of either 2 animals for each AAV serotype or 1 animal for PBS control. (**b**) At 14 days post AAV delivery the percentage of neurons positive for mCherry by immunohistochemistry were quantified in trigeminal ganglia using Cell Profiler software. Each symbol represents the average number of GFP positive neurons from 2 sections of an individual trigeminal ganglion from 3 different animals for each AAV serotype and PBS control. (**c**) Immunohistochemical detection of GFP and (**d**) hematoxylin/eosin staining of representative sections from trigeminal ganglia harvested at 14 days post AAV delivery. Scale bar: 100 μm.

Since AAV1 was able to efficiently traffic into the TG following whiskerpad delivery, we decided to see whether AAV serotypes that use different cellular receptors could also traffic to the TG. In addition to AAV1, AAV serotypes 5, 7, 8, and 9 have been used for gene delivery to neurons of the CNS and/or PNS^14, 16, 17, 19, 27^. These serotypes were therefore evaluated for their ability to deliver transgenes to TG neurons following intradermal whiskerpad injection (Fig. 2). scAAV-smCBA-GFP vectors of each serotype were delivered to the whiskerpad at a dose of 1 × 10^11^ AAV vector genomes per whiskerpad. Trigeminal ganglia were analyzed 7 days later by qPCR or 14 days later by immunohistochemistry (IHC) for levels of vector genomes and gene expression, respectively. qPCR analysis showed that regardless of the AAV serotype injected, similar levels of AAV vector genomes were detected per TG, demonstrating that AAV1, 5, 7, 8, and 9 trafficked from the whiskerpad to the TG with similar efficiency (Fig. 2a).

To initiate transgene expression, the viral particles that have trafficked to the neuronal cell bodies in the TG must enter the nucleus and uncoat. However, rates of nuclear import and capsid uncoating can vary in different cell types and by serotype^28, 29^. To confirm that AAV genomes could enter the nucleus and initiate transgene expression, IHC detection of the GFP transgene was performed on serial TG sections. The fraction of neurons expressing the transgene was determined using Cell Profiler Image Analysis software, which identifies neurons based on cell size, morphology, and neuronal marker (NeuN) staining. GFP staining for transgene expression (Fig. 2b,c, Supplemental Figure S1) and NeuN staining for neurons (data not shown) was performed on serial sections. All AAV serotypes except AAV5 expressed GFP in TG neurons, with AAV1 showing the highest percentage of GFP expression (Fig. 2b). Comparison against PBS treatment using Wilcoxon-Mann-Whitney test showed that AAV1, 7, 8, 9 (p = 0.05) were significantly different but not AAV5 (p = 0.8). Statistical analysis indicated that difference between groups is significant (p < 0.05, Krustal-Wallis rank sum test) and AAV1 is significantly different from any of the other AAV serotypes (p < 0.05, unpaired t-test). For all serotypes tested, GFP expression appeared to be restricted to neurons, with GFP immunoreactivity detected in both neuronal cell bodies and axons (Fig. 2c, Supplemental Figure S1), but not in the satellite glial cells surrounding neuronal cell bodies within the TG.

### AAV does not induce inflammation in the trigeminal ganglion

To determine whether any of the AAV serotypes induced inflammation in the trigeminal ganglion, we evaluated hematoxylin and eosin stained sections of TG collected from animals at 14 days post injection with PBS or 1 × 10^11^ vector genomes of scAAV-smCBA-GFP. Histological analysis did not reveal any evidence of immune infiltrates or tissue damage in the TG of any AAV treated mice when compared to PBS injected controls (Fig. 2d, Supplemental Figure S2).

### Transgene expression from constitutive and neuronal-specific promoters

**Figure 3:**
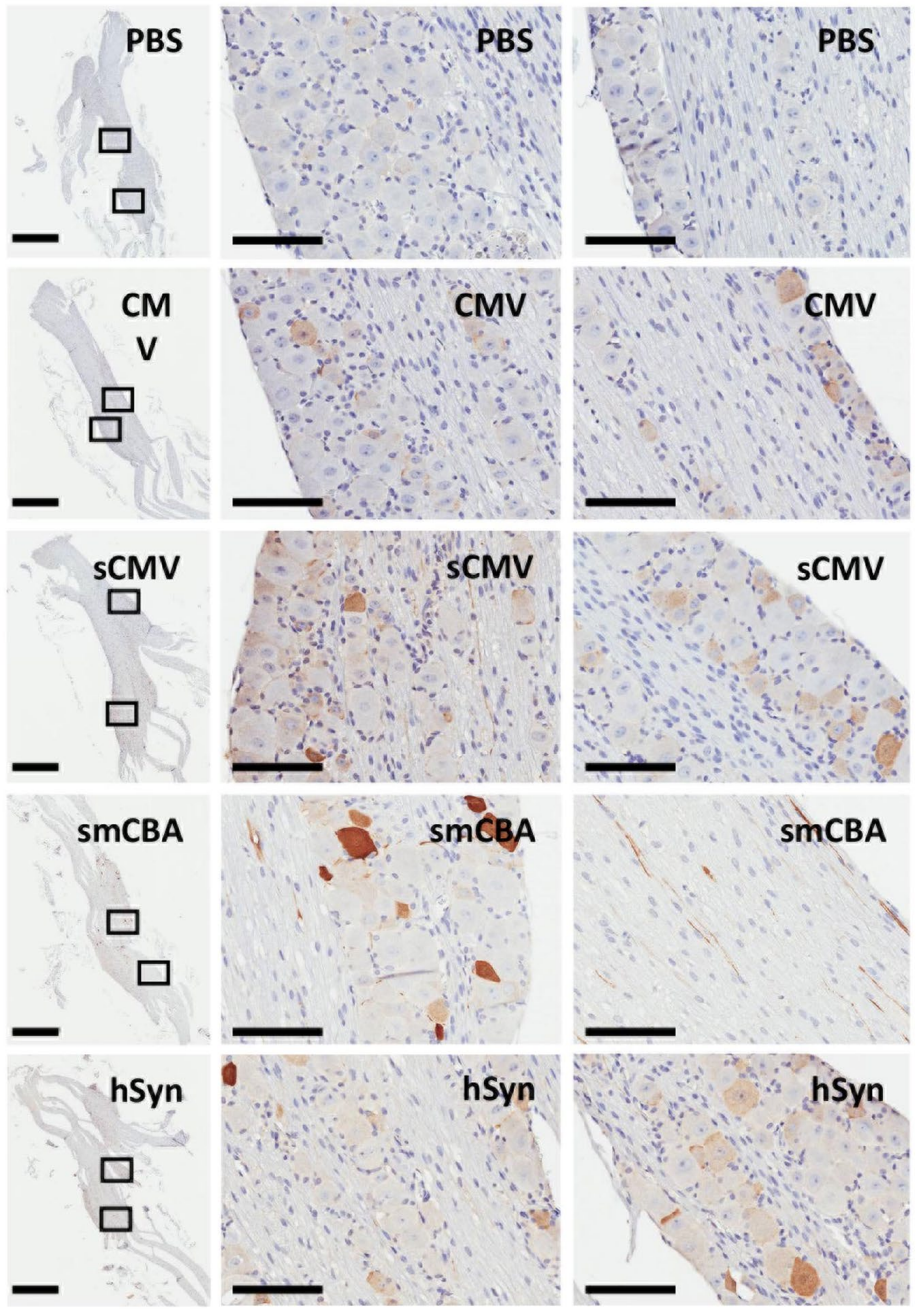
Comparison of constitutive and neuronal-specific promoters for gene expression in trigeminal ganglia. Swiss Webster mice were inoculated by intradermal injection of the whiskerpad with PBS or 1 × 10^11^ vector genomes of scAAV1-CMV-GFP, scAAV1-sCMV-GFP, scAAV1-smCBA-GFP or scAAV1-hSyn-GFP. Sections from trigeminal ganglia harvested at 14 days post AAV delivery were stained for GFP by immunohistochemistry. Scale bar: left panel - 1 mm, right two panels - 100 μm.

Previous experiments were performed using a shortened version of the constitutive hybrid CMV-chicken beta actin enhancer/promoter (smCBA). To test whether smCBA was the optimal promoter for transgene expression in TG neurons, other constitutive or neuronal-specific promoters were examined. AAV vector genomes with GFP under the control of the smCBA, CMV, sCMV (short CMV) or hSyn (human synapsin) promoters were packaged as scAAV1 vectors and delivered at a dose of 1 × 10^11^ vector genomes per whiskerpad. At day 14-post injection we analyzed GFP immunoreactivity in neurons following IHC staining of mice TG (Fig. 3). The TGs with the most intense GFP staining in both cell bodies and axons were from the mice injected with scAAV1-smCBA-GFP. The full length CMV promoter performed the worst and the sCMV and hSyn promoters drove an intermediate level of GFP expression.

### Levels of AAV1 genomes in the trigeminal ganglion persist for over 28 days post inoculation

**Figure 4:**
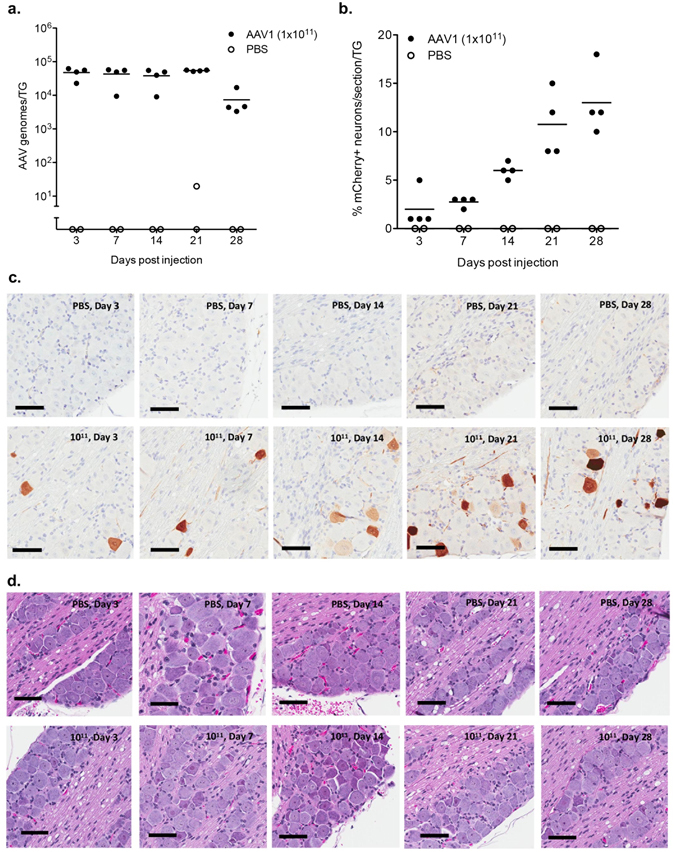
Longitudinal kinetics of scAAV1 gene delivery to trigeminal ganglia. Swiss Webster mice were inoculated by intradermal injection of the whiskerpad with PBS or 1 × 10^11^ vector genomes of scAAV1-smCBA-mCherry. At days 3, 7, 14, 21, and 28 post-delivery trigeminal ganglia were analyzed for levels of vector genomes by qPCR (**a**) or the percentage of neurons positive for mCherry by immunohistochemistry (**b**). For qPCR each symbol represents one trigeminal ganglion (TG) from a total of either 2 animals for AAV1 or 1 animal for PBS control at each time point. mCherry positive neurons were quantified using Cell Profiler software and each symbol represents the average number of mCherry positive neurons from 5 sections of one individual trigeminal ganglion (TG) from a total of either 2 animals for AAV1 or 1 animal for PBS control at each time point. Representative sections stained by immunohistochemistry for mCherry (**c**) or with hematoxylin/eosin (**d**) are shown from PBS or scAAV1-smCBA-mCherry injected mice. Scale bar: 100 μm.

To determine whether AAV vector genomes persist in the TG over time, we used qPCR to quantify AAV1 in the TG at 3, 7, 14, 21, and 28 days after whiskerpad injection of 1 × 10^11^ scAAV1-smCBA-mCherry vector genomes. We found AAV1 genomes in the TG by 3 days post-injection, which suggests that AAV1 vector entry into nerve terminals and retrograde transport into the TG occurs within that first 3-day window. Further, high numbers of AAV1 genomes persisted in the TG for at least 28 days (Fig. 4a). Statistical analysis indicated that the number of genomes per TG declined with time (p = 0.015, R^2^ = 0.25; linear regression of log-transformed AAV genomes/TG vs time). In a different experiment including a later time point, AAV genomes were still detected at high copy numbers at 56 days post injection; however, the levels of genomes showed a decline over time that was statistically significant (p = 0.0015, R^2^ = 0.75; linear regression of log-transformed AAV genomes/TG vs time; Supplemental Figure S3).

### AAV1-mediated transgene expression increases over 28 days following injection

Next, we analyzed the kinetics of transgene expression in transduced TG neurons. The proportion of TG neurons that expressed mCherry was analyzed by IHC at 3, 7, 14, 21 and 28 days post injection in the whiskerpad with 1 × 10^11^ vector genomes of scAAV1-smCBA-mCherry. We detected transgene expression as early as day 3, and over time the percentage of neurons that expressed mCherry continually increased through day 28 (Fig. 4b,c, Table 1, and Supplemental Figures S4 and S6). Statistical analysis indicated that the number of mCherry^+^ neurons per TG increased with time (p = 1.07 × 10^−7^, R^2^ = 0.79; linear regression of mCherry^+^ neurons/section/TG vs time). Histological analysis of hematoxylin- and eosin-stained sections showed no detectable inflammation or aberrant alterations in tissue morphology from day 3 through day 28-post inoculation of AAV1 when compared to PBS injected controls (Fig. 4d, Supplemental Figures S5 and S7).

**Table 1:** Percent transduction of TG neurons at increasing time post injection of 10^11^ AAV1 vector genomes.

Time of TG collection (days)	Mean % mCherry^+^ neurons	TG	Mean % mCherry^+^ neurons per TG	Range % mCherry^+^ neurons per section	% mCherry^+^ neurons per section for each TG
3	1.5	1	0.6	0.1–1.6	0.1–1.1–1.6–0.1–0.2
		2	0.8	0.4–1.3	0.4–0.9–0.5–1.3–0.9
		3	3.3	3.3–7.2	5.6–3.7–7.2–3.3–3.5
		4	1.2	0.6–2.1	1–2.1–0.6–0.9–1.6
7	2.6	1	3.4	2.3–5.3	3.8–3.1–2.3–5.3–2.3
		2	2.1	1.6–3.3	2.3–1.8–1.6–1.6–3.3
		3	2	0.7–4.4	4.4–1.3–0.7–1.3–2.3
		4	2.9	2.4–4.0	2.4–2.4–2.4–3.4–4.0
14	5.9	1	6.1	3.2–8.7	3.2–6.6–8.7–5.6–6.5
		2	6.9	4.2–8.0	7.5–4.2–8.0–7.3–7.6
		3	4.7	3.1–5.6	5.0–4.7–5.6–3.1–4.9
		4	6.0	4.4–8.4	4.4–6.1–5.4–8.4–5.8
21	10.7	1	14.6	10.6–23	10.6–11.0–13.0–15.3–23.0
		2	12.1	5.9–20.6	7.8–19.6–5.9–6.4–20.6
		3	8.3	6.5–9.8	8.4–8.9–6.5–7.8–9.8
		4	7.8	4.7–16.8	6.0–4.7–5.9–5.5–16.8
28	13.2	1	11.6	8.6–14.6	14.6–8.6
		2	18.1	18.1	18.1
		3	11.9	4.9–16.4	15.3–11.1–16.4–4.9
		4	10.5	7.5–18.0	18.0–9.4–7.1–7.5

### AAV1-mediated co-transduction of neurons within the trigeminal ganglion

**Figure 5:**
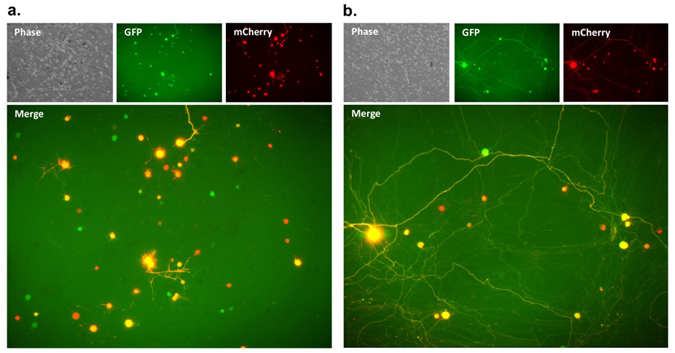
AAV co-transduction of sensory neurons in the trigeminal ganglia. (**a**,**b**) Swiss Webster mice were inoculated by intradermal injection of the whiskerpad with PBS or with 2 × 10^11^ vector genomes of scAAV1-smCBA-mCherry and 2 × 10^11^ vector genomes of scAAV1-smCBA-GFP. At 21 days post AAV delivery trigeminal ganglia were harvested and primary explant cultures were established to analyze numbers of mCherry and GFP positive cells by fluorescence microscopy. Representative images of primary sensory neuronal cultures are shown for 3 (**a**) and 29 days (**b**) post explant. For quantification GFP and mCherry positive neurons were counted in a total of 10 fields of view for each neuronal culture over 29 days.

One substantial limitation of AAV vectors is their small packaging capacity. Single-stranded AAV (ssAAV) vectors have a capacity of 4.7 Kb. This study was performed with self-complementary AAV (scAAV) vectors that typically give higher levels of transgene expression with faster kinetics when compared to ssAAV vectors^30, 31^. However, the packaging capacity of scAAV is half that of ssAAV, so the delivery of large genes or multiple genes is difficult. To partially overcome these hurdles, one can potentially co-transduce cells with multiple AAV vectors. We therefore asked whether we could co-transduce neurons in the TG via whiskerpad injection and express multiple transgenes in a single neuron. Mice were injected with both scAAV1-smCBA-mCherry and scAAV1-smCBA-GFP at a dose of 2 × 10^11^ vector genomes of each vector (total dose, 4 × 10^11^ vector genomes/whiskerpad) and were analyzed for the proportion of neurons that co-expressed mCherry and GFP at 21 days post injection. Since our IHC protocol does not allow us to distinguish between GFP and mCherry positive neurons, we quantified gene delivery to TG neurons by fluorescence microscopy using primary explant neuronal TG cultures. After harvesting TGs and establishing neuronal cultures, the percentage of neurons that expressed both reporter transgenes (GFP and mCherry) was calculated at 1, 2, 3, 4, 10, 18 and 29 days post explant. Double positive neurons were readily detected in explant cultures and the percentage of GFP/mCherry double-positive neurons did not change significantly over 29 days with an average of 57.1% (SD ± 12.2) of all reporter-expressing neurons expressing both GFP and mCherry (Fig. 5a,b, Supplemental Figure S8).

### Higher AAV doses lead to higher levels of gene delivery to the trigeminal ganglion

**Figure 6:**
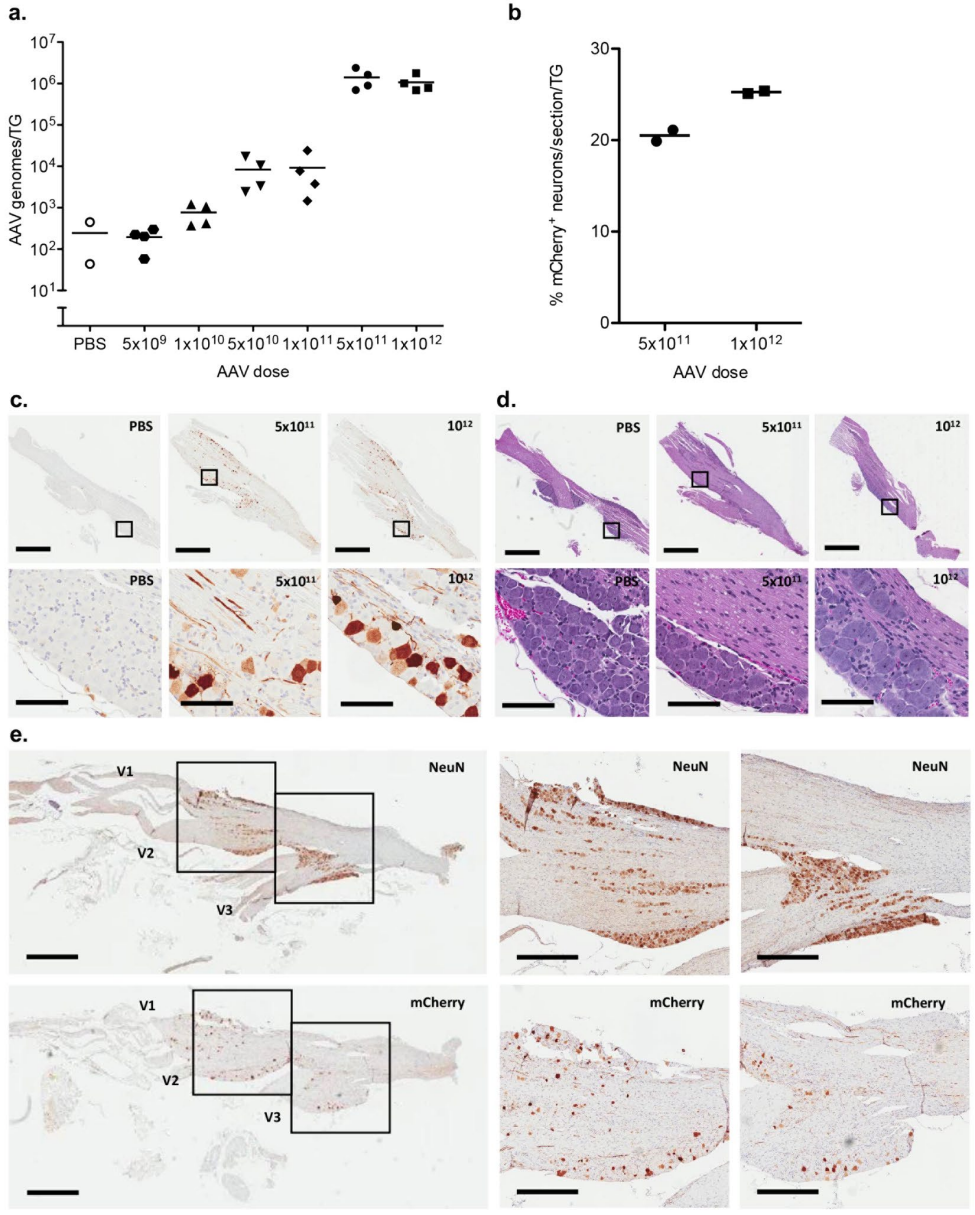
Dose optimization of scAAV1 gene delivery to trigeminal ganglia. Swiss Webster mice were inoculated by intradermal injection of the whiskerpad with PBS or increasing doses of scAAV1-smCBA-mCherry. At 6 weeks post AAV delivery trigeminal ganglia were analyzed for levels of vector genomes by qPCR (**a**). At the two highest doses the percentage of neurons positive for mCherry by immunohistochemistry were also determined (**b**). For qPCR each symbol represents one trigeminal ganglion (TG) from a total of either 2 animals for AAV1-treatment or 1 animal for PBS control for each dose. mCherry positive neurons were quantified using Cell Profiler software and each symbol represents the average number of mCherry positive neurons from 2 sections of one individual trigeminal ganglion (TG) from 1 animal for each dose. Representative sections stained by immunohistochemistry for mCherry (**c**) or with hematoxylin/eosin (**d**) are shown from PBS or scAAV1-smCBA-mCherry injected mice. Representative sections showing NeuN staining (Upper panel) or mCherry staining (Lower panel) in the V1 (Olfactory), V2 (Maxillary) and V3 (Mandibular) branches of the trigeminal ganglion are shown for animals that received 1 × 10^12^ vector genomes of scAAV1-smCBA-mCherry (**e**). Scale bars for **c**/**d**: upper panels - 1 mm, lower panels - 100 μm. Scale bars for **e**: left panel - 1 mm, right two panels - 500 μm.

To identify the AAV1 dose that gives the maximum levels of TG gene delivery we injected increasing doses of scAAV1-smCBA-mCherry (5 × 10^9^, 1 × 10^10^, 5 × 10^10^, 1 × 10^11^, 5 × 10^11^ and 1 × 10^12^ vector genomes) in the whiskerpad and quantified the levels of vector genomes in the TG at 7 days post injection by qPCR. We found that when the amount of AAV1 vector genomes injected in the whiskerpad was increased from 5 × 10^9^ to 1 × 10^12^, the levels of vector genomes in the TG increased from ~10^2^ to ~10^6^ genomes/TG. (Fig. 6a). We detected equivalently high transduction efficiency at injected doses of 5 × 10^11^ and 1 × 10^12^ AAV1 vector genomes (Fig. 6a). Statistical analysis indicated that the number of genomes per TG increased with time (p = 6.4 × 10^−13^, R^2^ = 0.9; linear regression of AAV genomes/TG vs dose, excluding PBS group).

We next evaluated the levels of AAV1 gene expression 6 weeks post whiskerpad delivery of scAAV1-smCBA-mCherry at doses of 5 × 10^11^ and 1 × 10^12^ vector genomes. We found an average of 20.5% and 25.25% mCherry positive neurons in the TG of these mice, respectively (Fig. 6b,c, Supplemental Figure S9). These data demonstrate that substantial levels of gene delivery can be achieved. Mice receiving 5 × 10^11^ or 1 × 10^12^ vector genomes were also analyzed for evidence of inflammation or alterations in tissue morphology. Hematoxylin and eosin stained sections from 6 weeks post injection showed no detectable inflammation or alterations in morphology when compared to PBS injected controls (Fig. 6d, Supplemental Figure S9).

### AAV1 delivers transgenes throughout the trigeminal ganglion

The whiskerpad is innervated by the maxillary nerve, one of the three branches of the TG. To determine whether neurons in other branches of the TG were also transduced, tissue sections from mice injected with 1 × 10^12^ vector genomes per whiskerpad were analyzed by IHC. A comparison of NeuN and mCherry stained TG sections from these mice demonstrated that the olfactory (V1), maxillary (V2) and mandibular (V3) branches of the trigeminal nerve all contained a substantial number of mCherry positive neurons (Fig. 6e). This widespread gene delivery to neurons throughout all branches of the TG should prove useful in the study of the craniofacial peripheral nervous system.

In a separate experiment, in which tissue sections from mice injected with 1 × 10^11^ vector genomes per whiskerpad were analyzed by IHC at day 7, 28 and 56, mCherry positive neurons were mainly observed in neurons near the roots of the TG branches at the earliest time point. By 28 days post injection, more distal neurons showed transgene expression while by day 56 mCherry^+^ neurons were readily observed at both proximal and distal regions of the root branch (Supplemental Figure S10).

## Discussion

We have investigated methods of peripheral AAV delivery as a tool to genetically manipulate sensory neurons of the TG. We found that the whiskerpad, which is innervated by afferent neurons projecting from the TG, is an efficient site for viral injection. We found that vectors based on AAV1 were the best serotype for gene delivery to TG neurons, and that we could deliver transgenes to up to 25% of neurons throughout the TG for a period of at least 6 weeks post delivery, without observable side effects.

Since previous studies have shown that HSV-1 can infect the TG after delivery to the scarified whiskerpad or cornea, we first determined whether AAV vectors delivered to the scarified cornea or whiskerpad dermis could also travel to neurons of the TG. Although we saw efficient uptake of AAV1 into the TG following intradermal whiskerpad delivery, we saw limited uptake after delivery to the scarified cornea. This latter result contrasts with recent data from Watson *et al*., who showed efficient transduction of rabbit TG neurons following ocular inoculation with AAV8 after corneal scarification^32^. In our own studies with AAV8 in mice, only limited uptake has been detected after ocular delivery to the scarified cornea (data not shown), and the biological reasons for this discrepancy are unclear. Unlike HSV-1, which is able to replicate in the eye and then traffic to neurons of the TG by retrograde transport along axons following uptake at ophthalmic (V1) nerve terminals, AAV vectors are non-replicating, and in the absence of replication after corneal delivery, AAV vectors may not be sufficiently exposed to nerve termini around the eye to enable efficient TG uptake. It is conceivable that localized delivery of AAV to the conjunctiva of the eye instead of the cornea would lead to more efficient transduction of the TG since the conjunctiva contains innervating nerve terminals from the ophthalmic nerve, although whiskerpad injection is likely the easier option.

Following whiskerpad delivery all AAV serotypes were detected in the TG at equivalent levels 7 days post delivery. This suggests that all serotypes are taken up at nerve termini and then traffic into the TG, presumably by retrograde transport along axons of the maxillary (V2) nerve. Based on the assumption that a mouse TG contains 20,000 to 40,000 neurons, our qPCR data suggests that this process enables uptake of approximately 2.5–5 AAV vector genomes into each neuron of the TG by day 7 post delivery for all serotypes following delivery of 1 × 10^11^ AAV vector genomes per whiskerpad. However, the method by which each serotype is taken up at peripheral nerve termini within the whiskerpad remains unknown. The AAV serotypes used in these studies can use unknown receptors (AAV7), or known receptors/co-receptors including N-linked sialic acids (AAV1 and AAV5), PDGFR (AAV5), lamininR (AAV8 and AAV9), or galactose (AAV9) for cell entry^29^. Intriguingly, there were equivalent levels of virus uptake into the TG from the whiskerpad for multiple different AAV serotypes suggesting that a receptor-independent pathway of neuronal uptake may be occurring. It is conceivable that upon whiskerpad delivery, AAV present in the vicinity of afferent neuronal projections in the whiskerpad dermis could be taken up into maxillary nerve termini during the process of presynaptic membrane retrieval and synaptic vesicle reformation^33^. Synaptic AAV uptake would subsequently enable it to travel into the TG through retrograde transport along axons, or through a transynaptic process as has been observed for the alphaherpesviruses HSV-1, VZV, and pseudorabies virus following initial replication in permissive non-neuronal cells^34–36^. Further studies will be needed to determine the mechanisms by which AAV can traffic from the whiskerpad dermis into the TG.

Despite the observation that AAV1, 5, 7, 8 and 9 all trafficked into the TG with equal efficiency, the levels of gene expression seen were highly variable, with AAV5 treated mice even showing a complete absence of expression in TG neurons. Of note, the AAV5 vector was not defective in gene expression, since cultured primary TG neurons showed transgene expression following transduction with the same AAV5 stock used in the present study^7^. The detection of equivalent levels of virus uptake into the TG from the whiskerpad for multiple different AAV serotypes could be attributed either to a receptor-independent pathway of neuronal uptake or to AAV serotypes using a common receptor during neuronal uptake such as the one recently reported by Pillay *et al*.^37^. These observations indicate that potential blocks to expression exist once a particular AAV serotype enters the TG. The rates of nuclear import^38^ and viral uncoating^28, 29^ can vary in different cell types depending on the AAV serotype, and this could be happening in sensory neurons of the TG. Furthermore, crucial cellular factors such as Cathepsins B and L that have been identified as AAV uncoating factors for AAV2 and AAV8^29^ may not be present in neurons of the TG, so that transgene expression cannot be initiated. The rate limiting steps for AAV expression in the TG are still to be defined.

Due to our selective delivery of AAV at a peripheral site that contains projections of TG neurons exclusively from the maxillary nerve, we initially hypothesized that gene expression in the TG might be localized to the cell bodies of neurons with afferent V2 projections into the whiskerpad. However, our analysis of gene expression at the highest doses of AAV tested showed that up to 25% of sensory neurons were positive for mCherry, with positive neurons seen throughout the V1, V2 and V3 regions of the TG. There are a number of possible explanations for this observation. First, AAV vectors might be able to traffic by trans-neuronal spread directly from V2 neurons into V1 or V3 neurons within the TG, as has been described for alphaherpesviruses^36^. Another explanation may be that the injection bolus is able to diffuse to other areas of the face where AAV may come into contact with ocular and mandibular nerve termini. Alternatively, the viral inoculum may leak into the blood stream from the injection site and subsequently enter the TG directly from circulating blood. Notably, a gradient effect was seen where the neurons located near the roots of the TG branches were the earliest to show transgene expression, while transgene expression in more distal neurons was more readily detected at later times post inoculation (Supplemental Figure S10a). It is worth noting that despite the transgene being under control of the constitutive smCBA promoter, expression was only detected in neurons and not the surrounding satellite glial cells, independent of AAV serotype (Fig. 2C, Supplemental Figure S1). This does not exclude the possibility that the satellite glials cells may be transduced but unable to express the transgene at detectable levels.

The comparison of different promoters showed that both constitutive and neuronal-specific promoters were able to drive transgene expression in TG neurons. However, the levels of transgene expression varied between promoters. One caveat is the analysis was performed at a single time point (14 days post delivery), and the promoters may exhibit different expression kinetics. Nonetheless, the shorter promoters provide an attractive alternative that would be suitable for packaging large transgenes into AAV.

Finally, we showed that following simultaneous injection of two AAV reporters at the periphery leads to at least 50% of the transduced TG neurons expressing the two transgenes delivered by the AAV vectors. This is consistent with our recent study^39^, in which we showed that when more than one AAV reporter is delivered, cells expressing one reporter gene are more likely than predicted by a random transduction model to also express the other reporter gene^39^.

In conclusion, we have performed an analysis of AAV gene transfer in the TG following intradermal delivery to the whiskerpad of Swiss Webster mice. We found that all AAV serotypes are able to efficiently traffic into the TG, but that AAV1 is the optimal serotype for gene expression within sensory TG neurons. Following AAV1 delivery, gene expression in neurons is dose dependent, increases over time, occurs throughout the TG, and does not induce inflammation or abnormal morphology within the TG. Taken together our data suggests that AAV1 could be an important tool for the study of neuropathic pain, peripheral nerve injury, or alphaherpesvirus infections.

## Methods

### Cell lines

HEK293^40^ and Vero cells (ATCC# CCL-81) were grown in DMEM (Invitrogen) with 10% FBS.

### AAV vector plasmids

Plasmids pscAAV-CMV-GFP and pscAAV-sCMV-GFP have been described previously^41^. pscAAV-CMV-mCherry was generated by PCR amplifying the mCherry gene with primers mCherry-F: GAGATCAGATCTCTCGAGGCCGCCACCATGGTGAGCAAGGGCGAGGAGGATAACATG and mCherry-R: GAGATCGCGGCCGCTCACTTGTACAGCTCGTCCATGCCGCCGGT, and cloning it into the BglII/NotI sites of pscAAV-CMV-pA^3^. pscAAV-smCBA-mCherry contains a small CBA/CAG promoter which has a truncated intron^42^. A PCR product containing the CBA enhancer/promoter and a segment of the truncated CBA intron was amplified by PCR from the plasmid pCAGGS-FLPe (Gene Bridges) using primers smCBA-F: GAGATCGGCGCGCCAATTCGGTACCCTAGTTATTAATAGTAATCAATTAC and Intron-R: GAGATCCTCGAGATGCATGAACATGGTTAGCAGAGGCTCTAGCTCCCGGAGCCCTTTAAGGCTTTCAC, and cloned into the AscI/XhoI sites of pscAAV-EFS-pA^43^ to generate pscAAV-smCBA-1. Then the mCherry gene fused to a segment of the CBA intron was amplified by PCR using primers Intron-F: GAGATCATGCATTCTTCTTTTTCCTACAGCTCCTGGGCAACGTGCTGGTTATTGTGCTGTCTCATCATTTTGGCAAAGGCGGCCGCGCCGCCACCATGGTGAGCAAGGGCGAGGAG and mCherry-R2: GAGATCGGATCCGTCGACAAGCTTTTAATTAAGCGGCCGCTCACTTGTACAGCTCGTCCATGCCGCCGGT, and cloned into the NsiI/BamHI sites of pscAAV-smCBA-1 to generate pscAAV-smCBA-mCherry. pscAAV-smCBA-GFP was generated by PCR amplifying the GFP gene using primers NotI-GFP-F: GAGATCGCGGCCGCGCCGCCACCATGGTGAGCAAGGGCGAGGAGCTGT and NotI-GFP-R: GAGATCGCGGCCGCTTACTTGTACAGCTCGTCCATGCCG, and substituting GFP with mCherry in pscAAV-smCBA-mCherry as a NotI fragment. pscAAV-hSyn-eGFP was generated by Gibson cloning a gBlock containing the human Synapsin I promoter (position 1889 to 2360 in sequence from Genbank M55301) flanked by AscI/BamHI sites (Sequence available upon request) into AscI/BamHI linearized pscAAV-CMV-GFP.

### AAV vector production

AAV vectors of all serotypes were generated by transiently transfecting 293 cells using PEI according to the method of Choi *et al*.^44^. Briefly, HEK293 cells were transfected with a scAAV vector plasmid, a plasmid that expresses the AAV rep and capsid proteins, and a helper plasmid that expresses adenovirus helper proteins (pHelper). At 24 hours post-transfection media was changed to serum-free DMEM and after 72 hours cells were collected and re-suspended in AAV lysis buffer (50 mM Tris, 150 mM NaCl, pH 8.5) before freeze-thawing 4 times. AAV stocks were purified by iodixanol gradient separation^44, 45^ followed by concentration into PBS using an Amicon Ultra-15 column (EMD Millipore) before storage at −80 °C.

### AAV tittering

All AAV vector stocks were quantified by qPCR using primers against the AAV ITR, with linearized plasmid DNA as a standard, according to the method of Aurnhammer *et al*.^46^. AAV stocks were treated with DNase I and Proteinase K prior to quantification. The titers calculated for all scAAV preps were determined without linearization of the AAV vector genome, which might result in underestimation of titers as previously described^47^. However, similar titers were obtained (data not shown) when scAAV titers were compared using the ITR-specific primers/probe and a previously described eGFP primers/probe set^48^ that amplifies a region distal to the scAAV genome central hairpin, a strategy that was previously shown to limit hairpin interference during titer determination^47^.

### Quantitation of AAV vector genomes in trigeminal ganglia

Trigeminal ganglia were surgically removed from mice and total DNA was extracted using the DNeasy Blood & Tissue Kit (Qiagen). Quantitation of AAV vector genomes in the trigeminal ganglion was performed by qPCR using AAV ITR-specific primers and probe^46^.

### Viral inoculation of mice

Mice were housed in accordance with institutional and NIH guidelines on the care and use of animals in research. All procedures were approved by the Institutional Animal Care and Use Committee of the Fred Hutchinson Cancer Research Center. The methods were carried out in accordance with the approved guidelines. Female Swiss Webster mice (Charles River), 4–8 weeks of age, were used for all studies. For ocular infections mice were exposed to viral inoculum with or without corneal scarification. Briefly, mice were anesthetized with Xylazine/Ketamine and then corneas were scarified with an 18-gauge needle by lightly scratching 5 times vertically and 5 times horizontally in a 5 × 5 grid. The indicated dose of virus was then pipetted onto the cornea in a 4 μl volume. For intradermal injection of the mouse whiskerpad mice were anesthetized with Xylazine/Ketamine, then injected with virus in a 50 μl volume using a 30-gauge needle insulin syringe. Intradermal whiskerpad injection of PBS or AAV produced a bolus at the injection site that resolved to normal within 24 hours of inoculation.

### Culture of primary neurons from mouse trigeminal ganglion

Mouse trigeminal ganglia were surgically harvested at the indicated time following euthanasia with CO_2_. Neuronal cultures were established after enzymatic digest as described by Bertke *et al*.^49^. Neurons were counted and plated on poly-D-lysine- and laminin-coated 6-well plates (BD Biosciences). Neuronal cultures were maintained with complete neuronal medium, consisting of Neurobasal A medium supplemented with 2% B27 supplement, 1% PenStrep, L-glutamine (500 μM), and nerve growth factor (50 ng/ml). Medium was changed every 2–3 days.

### Microscopy

Fluorescence signals (Fig. 5) were imaged by direct visualization using a TE2000 microscope (Nikon) equipped with a CDD camera (CoolSNAP ES, Photometrics) and Metavue software (Universal imaging).

### Immunohistochemistry

Trigeminal ganglia were fixed in 10% neutral buffered formalin, paraffin embedded then sectioned at 4 μm thickness. For mCherry staining, antigen retrieval was performed for 12 hours at pH 9 in a 65 °C water bath using Tris-EDTA buffer. Sections were then stained with a mouse monoclonal anti-mCherry antibody (1:1000) provided by FHCRC Experimental Histopathology (Benjamin Hoffstrom) and detection was performed using PowerVision poly-HRP anti-mouse (Leica) along with the chromagen DAB (Dako) 2 times for 4 minutes. Slides were then counterstained with hematoxylin (Biocare) and Tacha’s bluing solution at 25% for 2 minutes. Isotype controls of mouse Ig were used as negative control. For GFP staining, antigen retrieval was performed as above for 35 min at 65 °C. Sections were then stained with a rabbit polyclonal anti-GFP antibody (A11122 Invitrogen 1:1000) and detection was performed with using PowerVision poly-HRP anti-rabbit, (Leica) along with the chromagen DAB for 10 minutes. Slides were then counterstained with hematoxylin 50% (Biocare NM-HEM-M) for 3 minutes. Rabbit immunoglobulin was used as negative control. For NeuN staining, antigen retrieval was performed as above for 12 hours at pH9 in a 65 °C water bath. Sections were then stained with a mouse monoclonal anti-NeuN antibody (clone A60, Millipore mab377, 1:800), and detection was performed using PowerVision poly-HRP anti-rabbit and the Mouse on Mouse Basic Kit (Vector Laboratories, BMK-2202).

### Image quantification

Quantification of mCherry or GFP positive neurons in sections of mouse TG was performed using CellProfiler software as previously described^50^. Briefly, DAB stained bright field images were scanned at 40X (Aspera) and captured at fixed exposure and position using ImageScope software. Computer cell counting required grey scale images with bright areas of interest. The mCherry, GFP and NeuN stained images were split into blue, red, and green channels and inverted. The brightness of desired objects was highest over background in the blue channel, so the blue channel was analyzed in CellProfiler^50^. This program produced cells counts for the images and also saved an image with the counted cells outlined for quality control inspection. Counts due to artifacts (tears, edges, folds) were subtracted from the computer counts to yield the final count.

## Electronic supplementary material

Supplementary Figures
