# Supplementary material for: In vivo dynamics of AAV-mediated gene delivery to sensory neurons of the trigeminal ganglia
Source: Sci Rep. 2017 Apr 19;7:927. doi: 10.1038/s41598-017-01004-y (PMC5430444; doi:10.1038/s41598-017-01004-y)
Supplement: Supplementary file 1 — Supplementary Figures [file 41598_2017_1004_MOESM1_ESM.pdf]

In vivo dynamics of AAV-mediated gene delivery to sensory neurons of the trigeminal ganglia

Chung H. Dang, Martine Aubert, Harshana S. De Silva Feelixge, Kurt Diem, Michelle A. Loprieno, Pavitra Roychoudhury, Daniel Stone and Keith R. Jerome

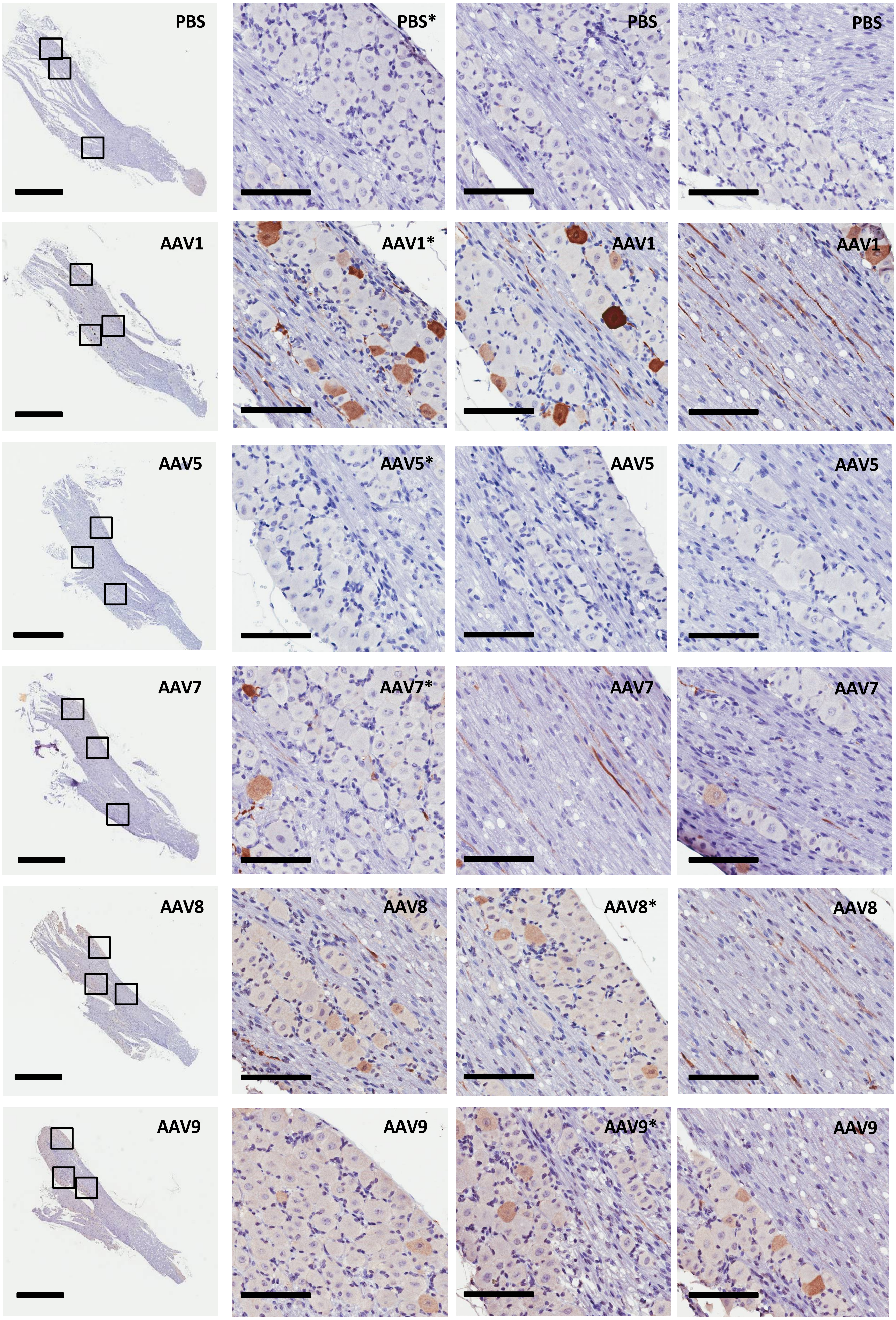

Supplemental Figure 1

Supplemental Figure 1. Representative images of TG sections stained for mCherry by immunohistochemistry from the experiment described in Figure 2. Mice received PBS or scAAV-smCBA-GFP vectors packaged into AAV serotypes 1, 5, 7, 8 or 9 at a dose of 1 x 10^{11} vector genomes per whiskerpad and trigeminal ganglia were harvested at 14 days post injection for analysis. Scale bars: left panels - 1mm, right three panels - 100μm.

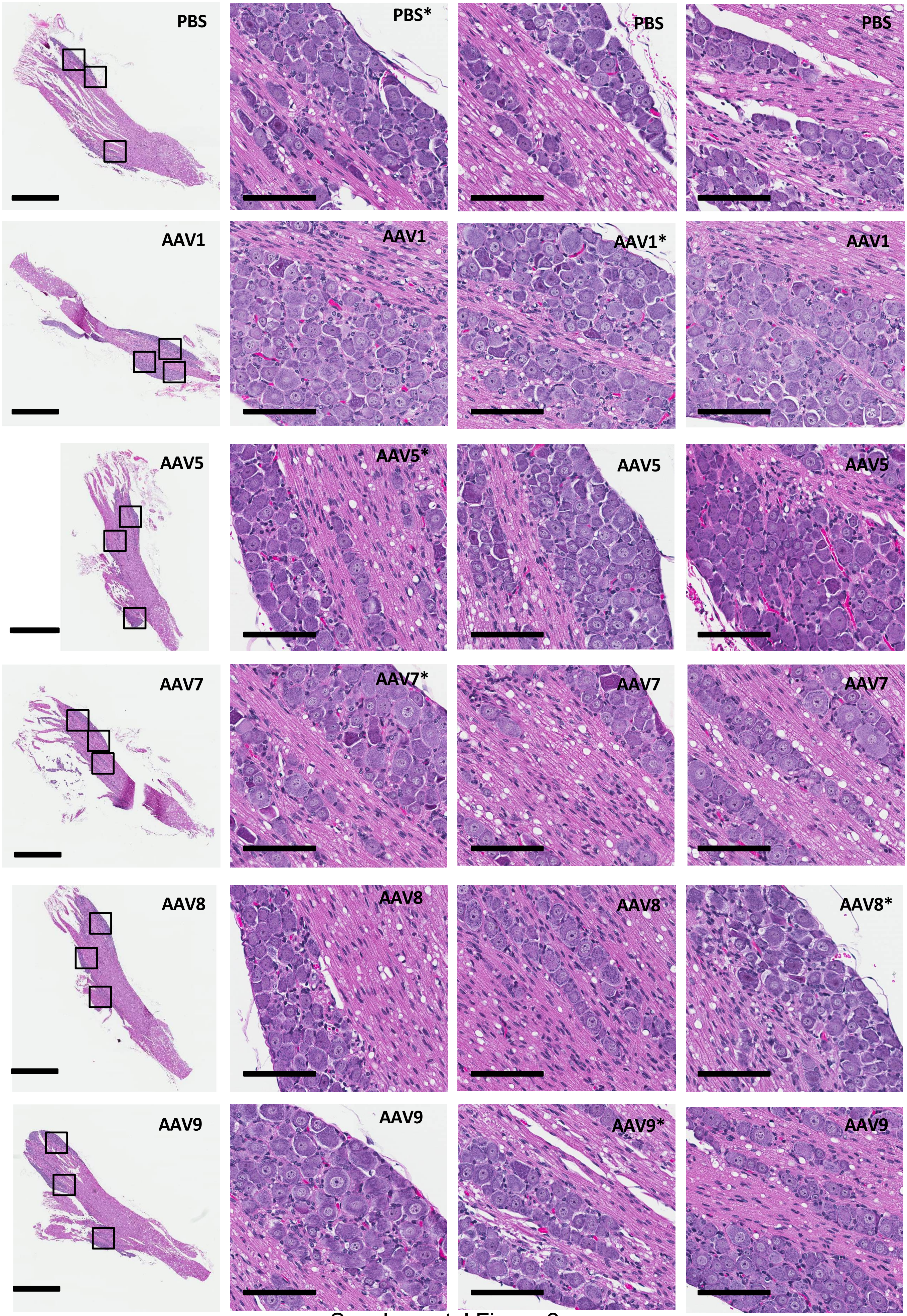

Supplemental Figure 2

Supplemental Figure 2. Representative images of TG sections stained with hematoxylin/eosin from the experiment described in Figure 2. Mice received PBS or scAAV-smCBA-GFP vectors packaged into AAV serotypes 1, 5, 7, 8 or 9 at a dose of 1 x 10^{11} vector genomes per whiskerpad and trigeminal ganglia were harvested at 14 days post injection for analysis. Scale bars: left panels - 1mm, right three panels - 100μm.

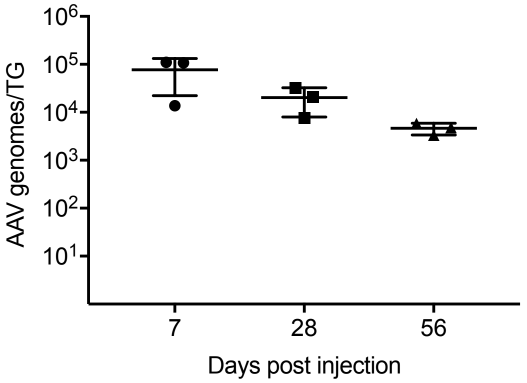

Supplemental Figure 3

Supplemental Figure 3. Persistence of scAAV1 genomes in trigeminal ganglia. Swiss Webster mice were inoculated by intradermal injection of the whiskerpad with 1 x 10^{11} vector genomes of scAAV1-smCBA-mCherry. At days 7, 28, ad 56 post delivery, one trigeminal ganglia from three different mice per time point were analyzed for levels of vector genomes by ddPCR.

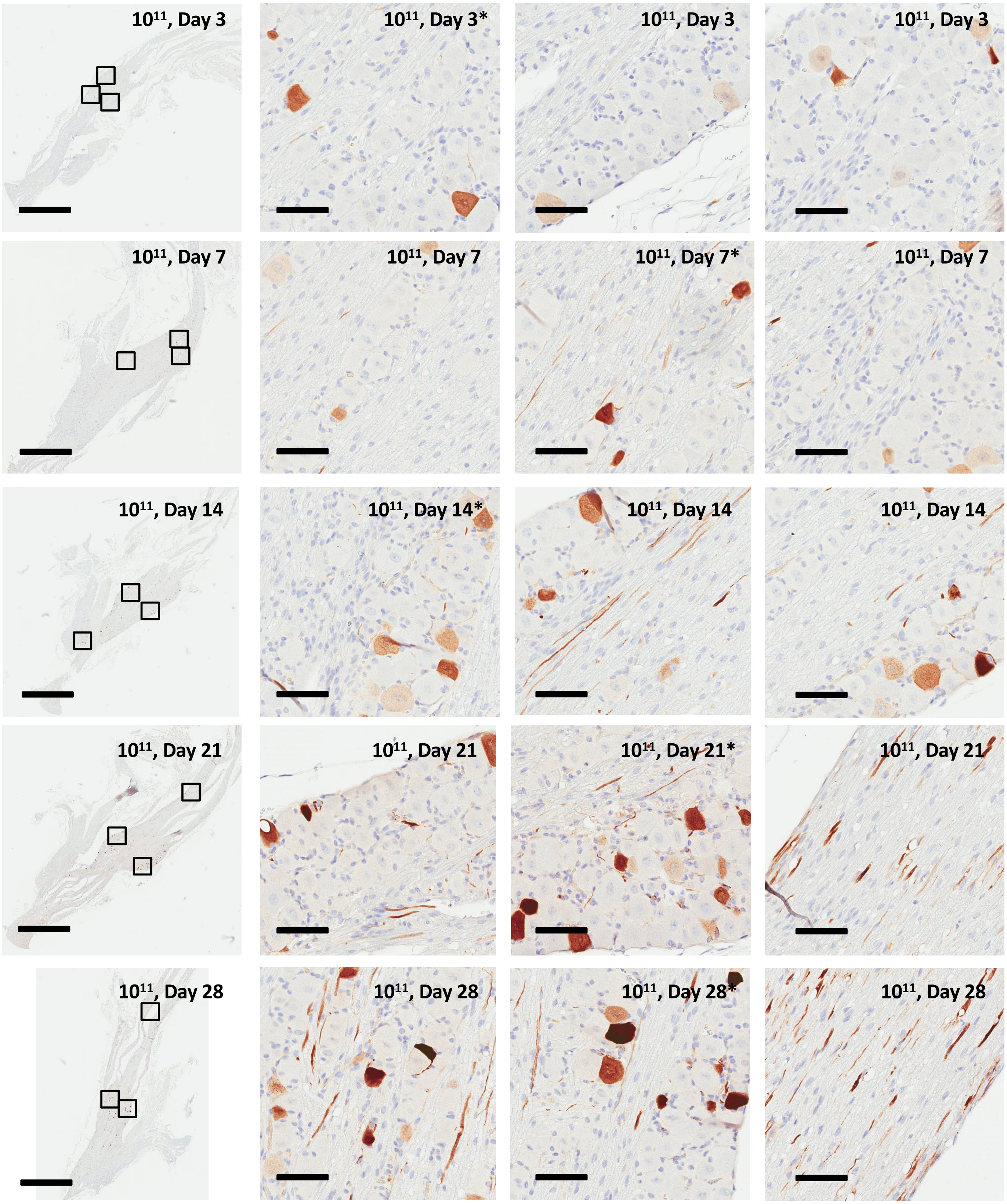

Supplemental Figure 4

Supplemental Figure 4. Representative images of TG sections stained for mCherry by immunohistochemistry from the experiment described in Figure 4. Mice received scAAV1-smCBA-GFP vector at a dose of 1 x 10^{11} vector genomes per whiskerpad and trigeminal ganglia were harvested at 3, 7, 14, 21 and 28 days post injection for analysis. Scale bars: left panels - 1mm, right three panels - 100μm.

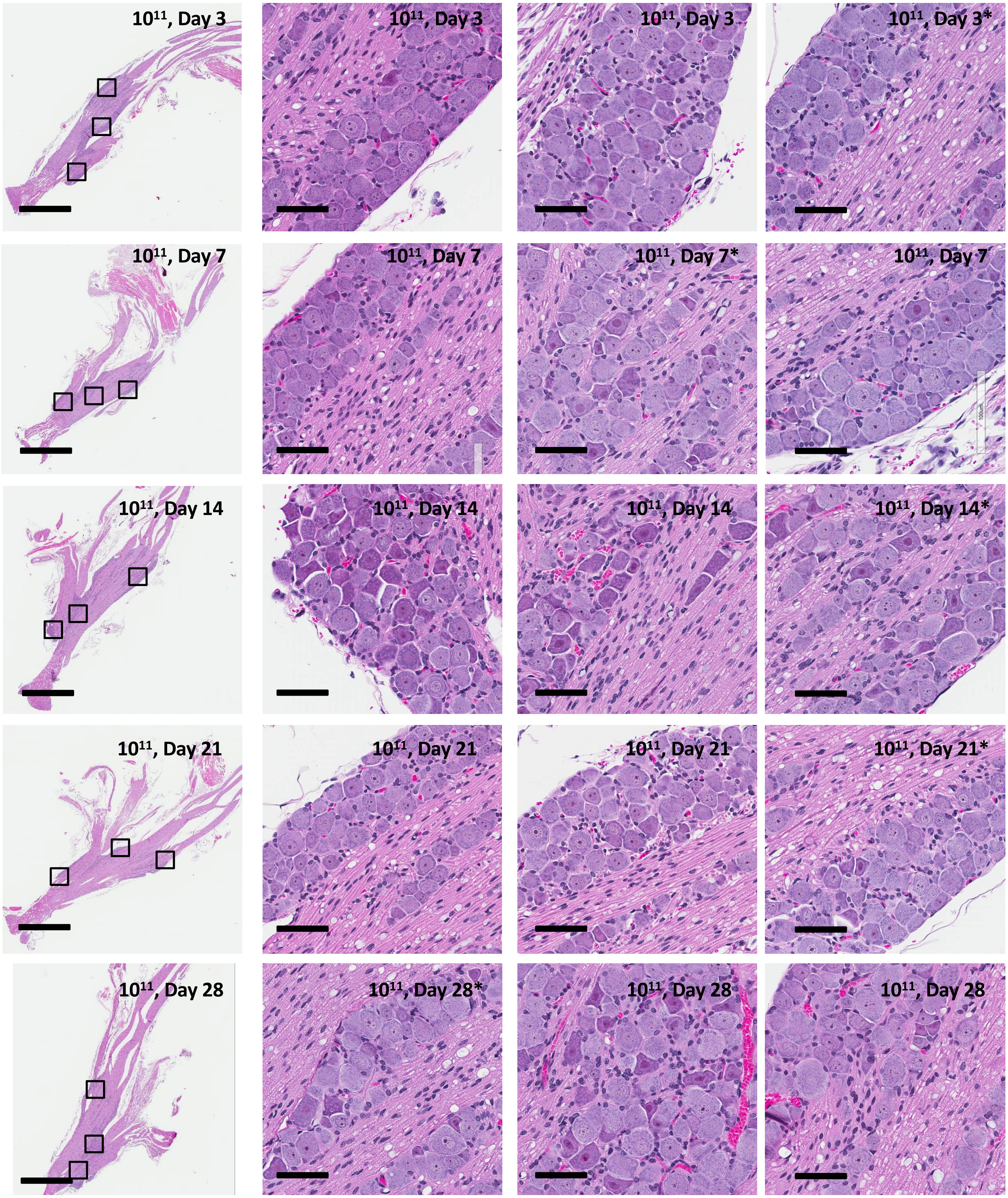

Supplemental Figure 5

Supplemental Figure 5. Representative images of TG sections stained with hematoxylin/eosin from the experiment described in Figure 4. Mice received scAAV1-smCBA-GFP vector at a dose of 1 x 10^{11} vector genomes per whiskerpad and trigeminal ganglia were harvested at 3, 7, 14, 21 and 28 days post injection for analysis. Scale bars: left panels - 1mm, right three panels - 100μm.

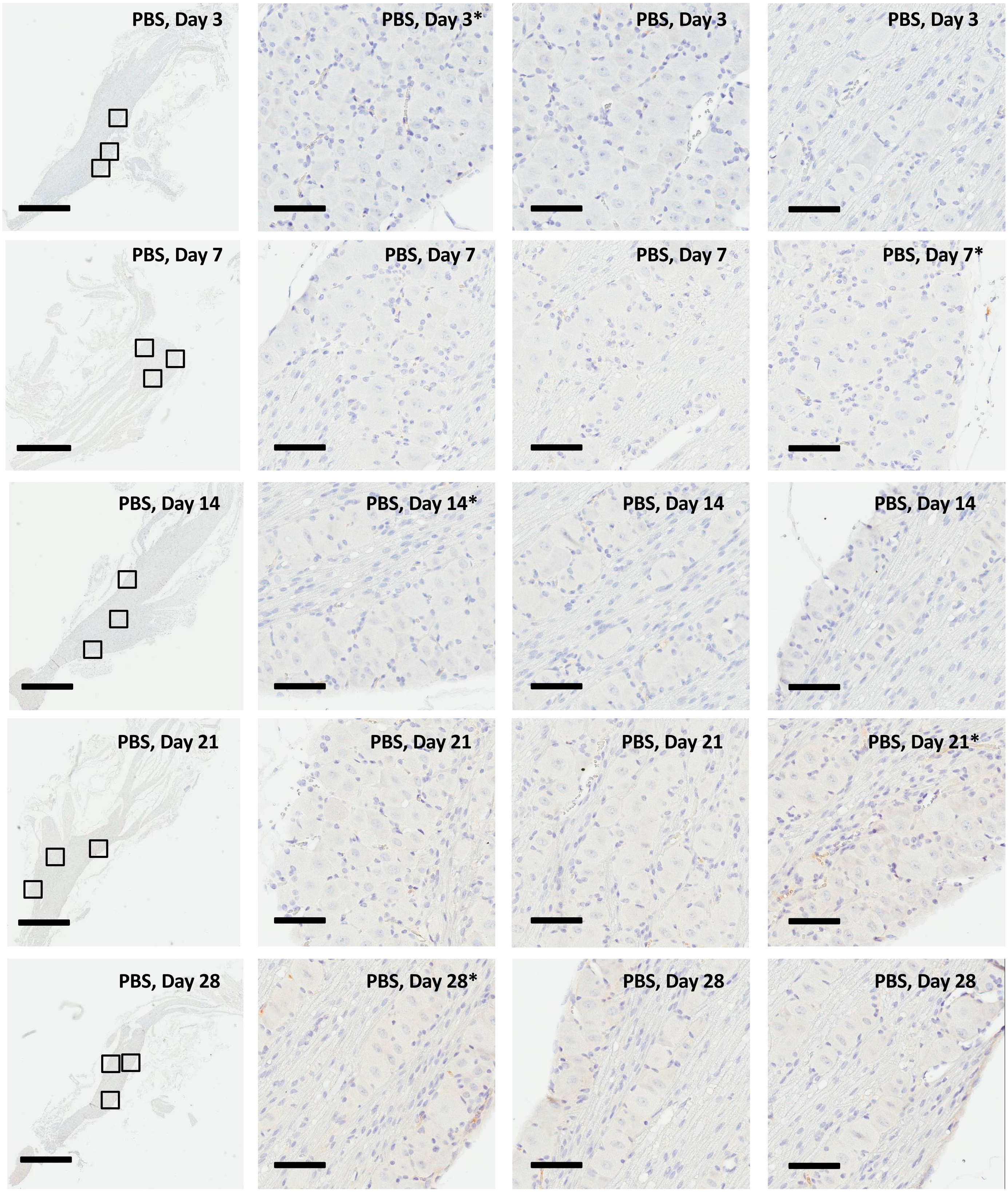

Supplemental Figure 6

Supplemental Figure 6. Representative images of control TG sections stained for mCherry by immunohistochemistry from the experiment described in Figure 4. Mice were injected in the whiskerpad with PBS and trigeminal ganglia were harvested at 3, 7, 14, 21 and 28 days post injection for analysis. Scale bars: left panels - 1mm, right three panels - 100μm.

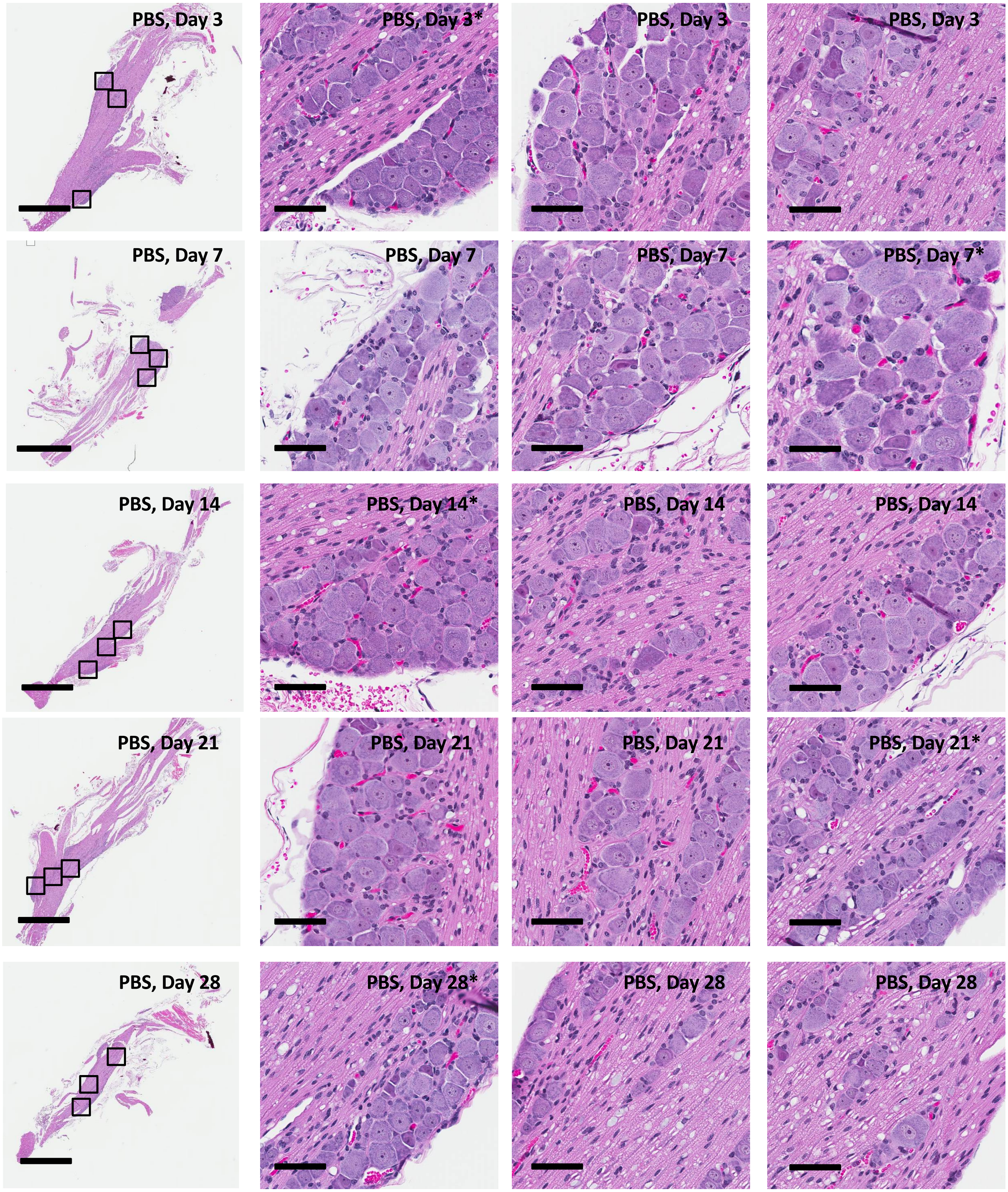

Supplemental Figure 7

Supplemental Figure 7. Representative images of control TG sections stained with hematoxylin/eosin from the experiment described in Figure 4. Mice were injected in the whiskerpad with PBS and trigeminal ganglia were harvested at 3, 7, 14, 21 and 28 days post injection for analysis. Scale bars: left panels - 1mm, right three panels - 100μm.

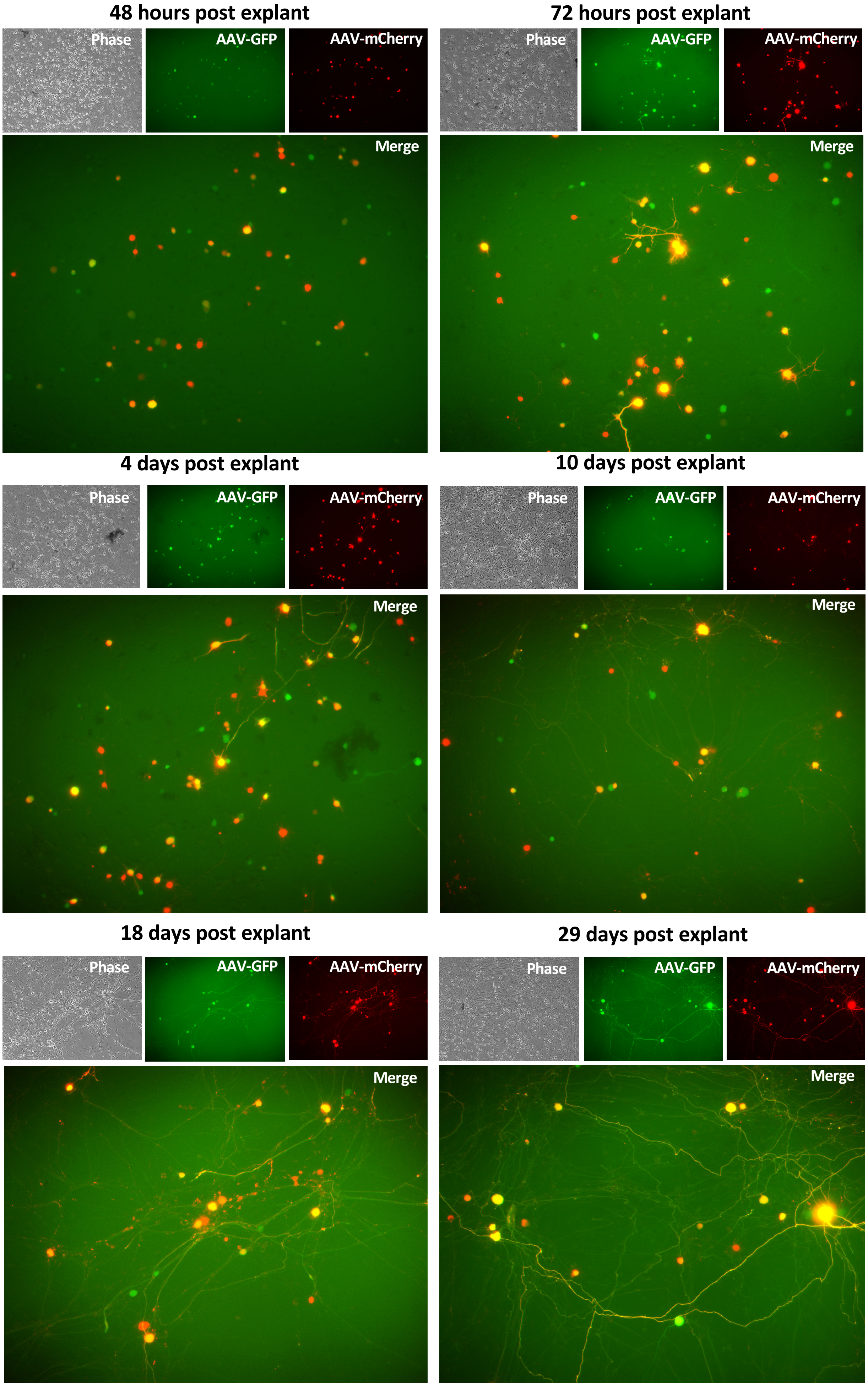

Supplemental Figure 8

Supplemental Figure 8. Phase and fluorescent microscopy images of primary sensory neuronal explant cultures from the experiment described in Figure 5. Mice were inoculated by intradermal injection of the whiskerpad with PBS or with 2 x 10^{11} vector genomes of scAAV1-smCBA-mCherry and 2 x 10^{11} vector genomes scAAV1-smCBA-GFP. At day 21 post AAV delivery trigeminal ganglia were extracted and primary explant cultures were established. Representative images of primary sensory neuronal cultures are shown after 2, 3, 4, 10, 18 and 29 days in culture.

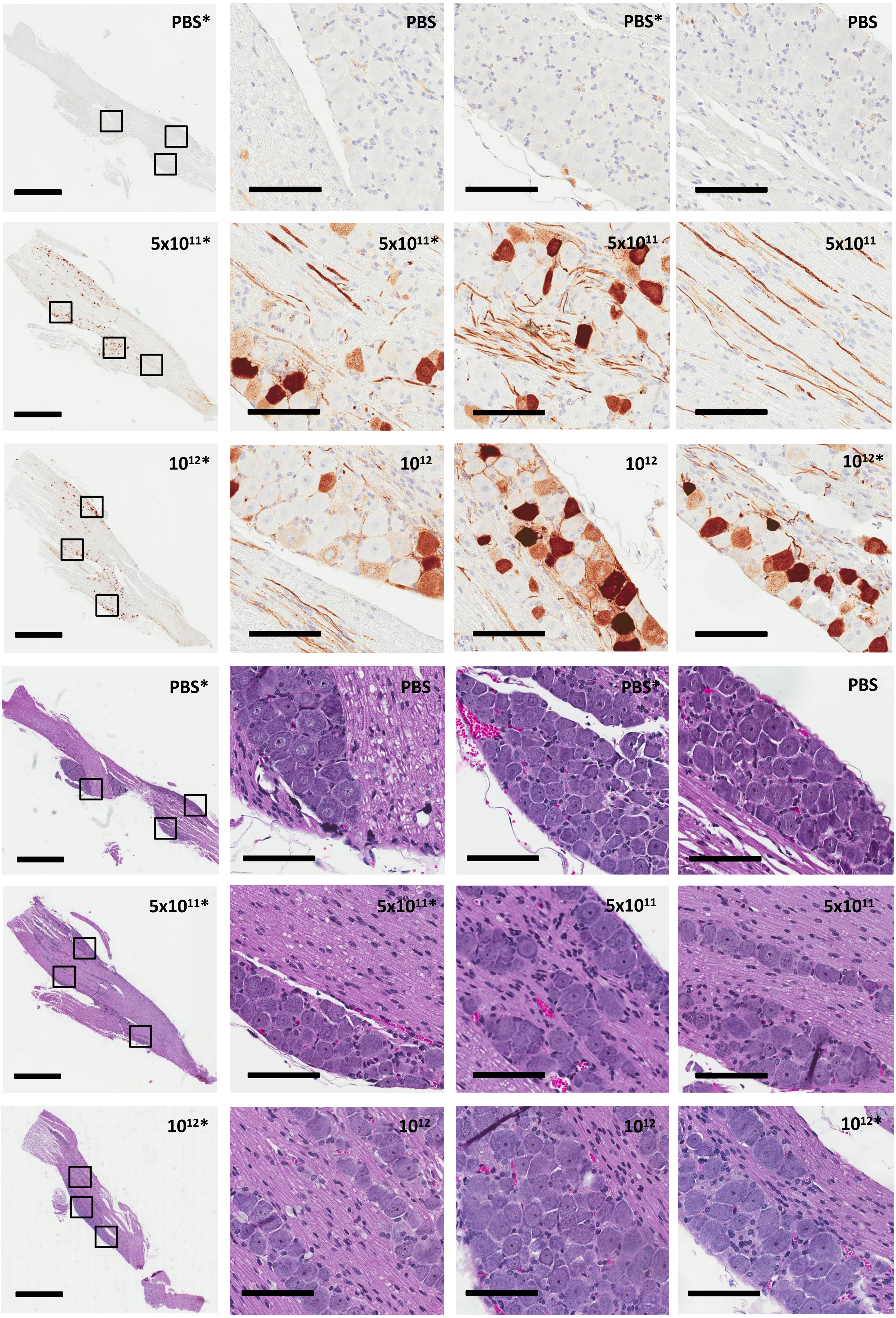

Supplemental Figure 9

Supplemental Figure 9. Representative images of TG sections stained for mCherry by immunohistochemistry (upper three panels) or with hematoxylin/eosin (lower three panels) from the experiment described in Figure 6. Mice received PBS or scAAV1-smCBA-GFP vector at a dose of 5 x 10^{11} or 1 x 10^{12} vector genomes per whiskerpad and trigeminal ganglia were harvested at 6 weeks post injection for analysis. Scale bars: left panel - 1mm, right three panels - 200μm.

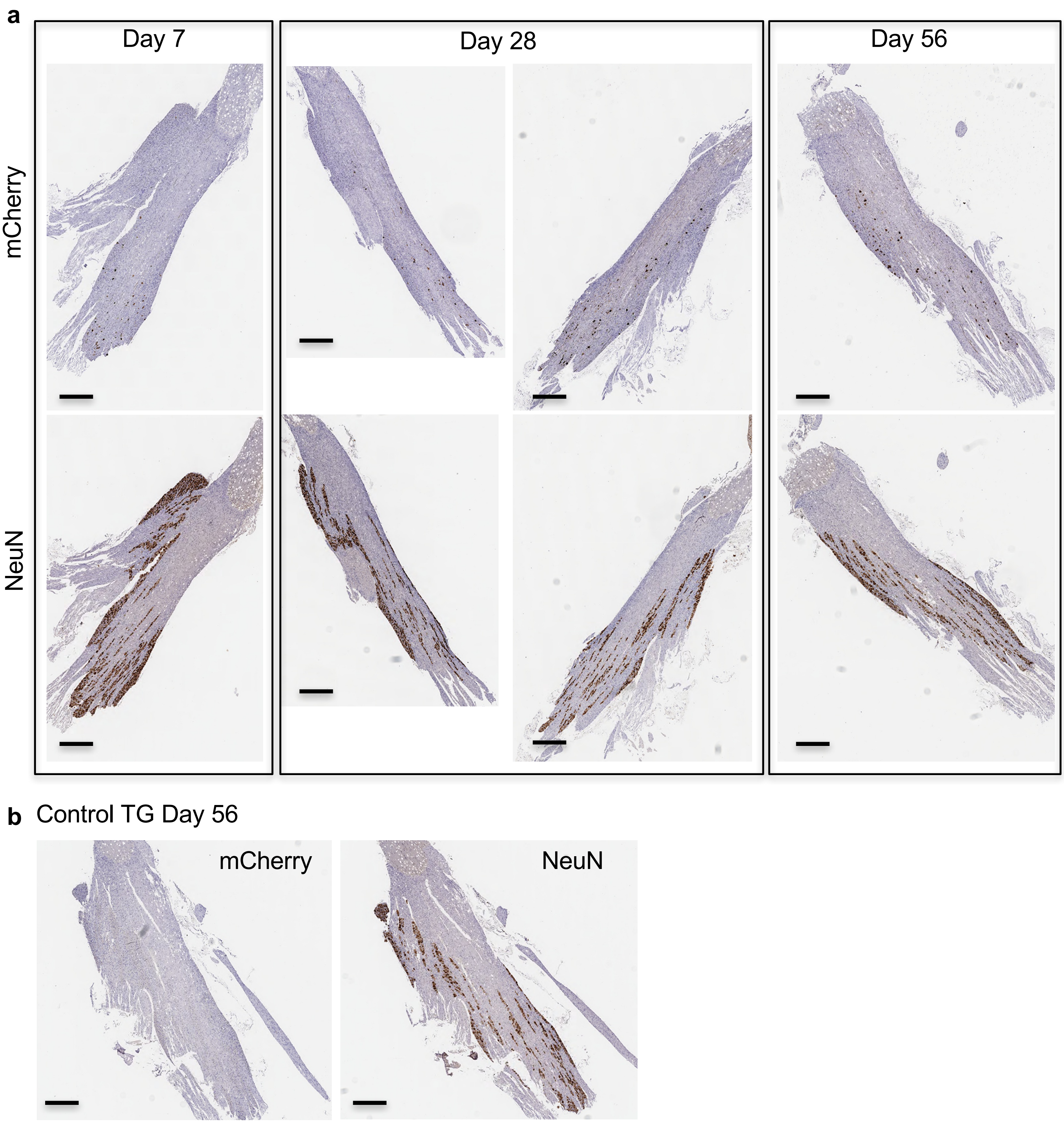

Supplemental Figure 10

Supplemental Figure 10. Representative images of TG sections stained for mCherry or NeuN by immunohistochemistry from the experiment described in Supplemental Figure 3. TG sections obtained from mice injected with 1×10^{11} AAV1 vectors (**a**) or PBS (**b**). Scale bars: 400μm.

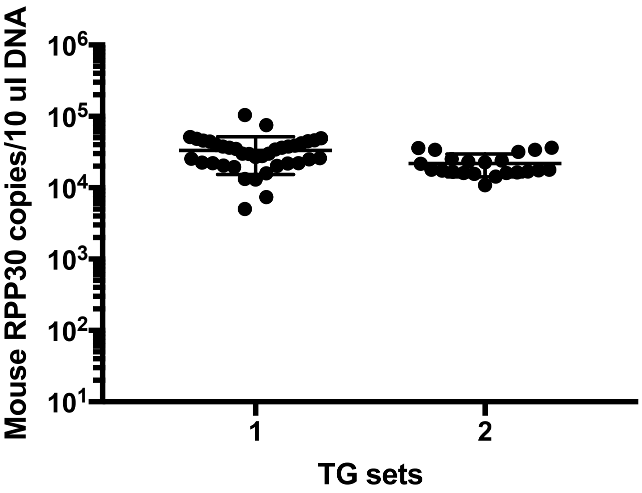

Supplemental Figure 11

Supplemental Figure 11. Quantification of total DNA obtained from TG. The quantification of the endogenous cellular genome was done using primer/probe set specific for the mouse housekeeping gene RPP30. Each dots in the graph represents the quantity of endogenous cellular genomes from one TG in two independents sets of animals: Set 1; 38 mice and Set 2; 22 mice.
